# Enhanced Luminescence of Yb^3+^ Ions Implanted to ZnO through the Selection of Optimal Implantation and Annealing Conditions

**DOI:** 10.3390/ma16051756

**Published:** 2023-02-21

**Authors:** Renata Ratajczak, Elzbieta Guziewicz, Slawomir Prucnal, Cyprian Mieszczynski, Przemysław Jozwik, Marek Barlak, Svitlana Romaniuk, Sylwia Gieraltowska, Wojciech Wozniak, René Heller, Ulrich Kentsch, Stefan Facsko

**Affiliations:** 1 National Centre for Nuclear Research, Soltana 7, 05-400 Otwock, Poland; 2 Institute of Physics, Polish Academy of Sciences, Al. Lotnikow 32/46, 02-668 Warsaw, Poland; 3 Helmholtz-Zentrum Dresden-Rossendorf, Institute of Ion Beam Physics and Materials Research, Bautzner Landstrasse 400, D-01328 Dresden, Germany

**Keywords:** wide bandgap oxides, zinc oxide, rare earth, ion implantation, annealing, RTA, FLA, PPA, Rutherford backscattering spectrometry, RT-photoluminescence

## Abstract

Rare earth-doped zinc oxide (ZnO:RE) systems are attractive for future optoelectronic devices such as phosphors, displays, and LEDs with emission in the visible spectral range, working even in a radiation-intense environment. The technology of these systems is currently under development, opening up new fields of application due to the low-cost production. Ion implantation is a very promising technique to incorporate rare-earth dopants into ZnO. However, the ballistic nature of this process makes the use of annealing essential. The selection of implantation parameters, as well as post-implantation annealing, turns out to be non-trivial because they determine the luminous efficiency of the ZnO:RE system. This paper presents a comprehensive study of the optimal implantation and annealing conditions, ensuring the most efficient luminescence of RE^3+^ ions in the ZnO matrix. Deep and shallow implantations, implantations performed at high and room temperature with various fluencies, as well as a range of post-RT implantation annealing processes are tested: rapid thermal annealing (minute duration) under different temperatures, times, and atmospheres (O_2_, N_2_, and Ar), flash lamp annealing (millisecond duration) and pulse plasma annealing (microsecond duration). It is shown that the highest luminescence efficiency of RE^3+^ is obtained for the shallow implantation at RT with the optimal fluence of 1.0 × 10^15^ RE ions/cm^2^ followed by a 10 min annealing in oxygen at 800 °C, and the light emission from such a ZnO:RE system is so bright that can be observed with the naked eye.

## 1. Introduction

Zinc oxide (ZnO) is a promising transparent material, which belongs to the group of wide bandgap (WBG) semiconductors. Its bandgap (~3.37 eV at T = 300 K) is very close to that of gallium nitride (GaN ~3.39 eV), currently leading in device production, but the technology of growing ZnO crystals and films is much easier and cheaper than GaN.

The unique properties of this material place ZnO in the frontline of future applications in electronics (Schottky junctions dedicated to a new type of non-volatile crossbar memories, conductive electrodes, ultrasonic transducers, spintronic, and organic electronics) and sensing systems (biosensors, UV and nuclear radiation detectors) [1,2]. However, the high exciton binding energy (~60 meV at room temperature (RT), thus 2.4 times higher than that of GaN) predestinates ZnO for unrivaled optical and optoelectronic applications in phosphors, light-emitting diodes (LED), and display technology [3,4,5,6,7]. ZnO is also more radiation resistant compared to GaN [8].

The fundamental near-band emission from ZnO is located in the ultraviolet spectral range. However, doping with rare-earth (RE) metals can tune the optical emission into the visible spectral range [9,10,11,12,13]. Moreover, it is expected that due to its high bandgap, ZnO can be an especially efficient luminescent host material for a number of trivalent rare-earth ions, overcoming the temperature-quenching effect observed in other materials used as RE matrices (e.g., in Si) [14,15]. Therefore, the ZnO:RE systems seem to be attractive for future optoelectronic devices with emissions in the visible region working even in radiation-intense environments.

Implantation of RE ions provides precise control of the concentration and depth distribution of dopants and hence, it is a very promising technique for the production of such systems [16]. However, an important limitation of this technique is the build-up of lattice disorder due to the ballistic nature of the process. Typically, structural defects are undesirable because they adversely affect the lifetime of devices based on defective materials. On the other hand, they quite often act as emission traps, strongly influencing luminescence [17,18,19].

In the last few years, several papers on heavy ion-implanted ZnO have been published [9,17,20,21,22,23,24,25,26,27,28,29,30]. It has been established that ZnO cannot be driven amorphous even after heavy ion bombardment with fluences of up to 100 displacements per atom (DPA) [9]. The maximum level of produced damage exhibits negligible dependence on the ion beam flux [22], collision cascade density [23], and sample temperature in the range of 20–400 °C [24]. However, the anisotropy of ZnO irradiated with Gd ions has also been reported [25,26]. The effect of the RE dopant accumulation on the structure and defects of the crystal lattice as well as the neighborhood environment of Yb ions has been already reported [28,29,30,31]. It was found that in the first stage, nuclear collisions of penetrating ions with matrix atoms create mainly simple defects, such as vacancies and interstitials. Further accumulation of point defects upon increasing the ion fluence leads to the creation of different types of extended structural defects and their transformation into more complicated forms, making the accumulation process in ion-implanted compound crystals a multi-step phenomenon [28,29]. The HRXRD studies carried out to date [27,28] clearly show that the reason for the defect transformations is the tensile strain that grows up with an increase in ion fluence, according to Hooke’s law. When the critical yield of the stress is attained, then the plastic deformation due to dislocation slips takes place. Consequently, the stress is released, the dislocations become mobile and the defect structure transformation to a new form occurs. Additionally, the depth profile of the built-up damage in the ZnO lattice is atypical and proceeds differently than in other semiconductor compounds, e.g., in GaN [32,33]. The latter finding is supported by channeling Rutherford backscattering spectrometry (RBS/c) analysis [34,35], which clearly shows an additional damage peak in the aligned spectra [36,37,38].

It should be emphasized that in the case of ZnO implanted with RE ions at standard conditions (RT), most of the RE dopants remain optically inactive at the as-implanted stage, regardless of the fluences used [39]. Thus, post-implantation annealing has to be applied to induce structural recovery and optically activate the implanted dopants [39,40,41,42,43,44].

However, it has been demonstrated that the annealing process can only be successful for implantation fluences below the critical one, i.e., below the threshold of plastic deformation. The plastic deformation threshold for ZnO:RE systems was estimated at 1.5 × 10^15^ RE ions/cm^2^, which corresponds to the values of ~8 DPA and ~0.14 at. % [29]. Above this critical fluence, extended defects are formed, such as dislocation tangles and stacking faults [28]. A crystal mosaic [45] and large defect clusters related to RE and native defects of ZnO can also be observed [17,30,31,46,47]. It should be noted that such new forms of the defect and defect complexes, which form in the matrix for ion fluences higher than the plastic deformation threshold, are resistant to post-implantation thermal annealing [30,48].

Optical and electrical properties are strongly related to the threshold of structural transformation. It has been found that two effects take place at this threshold: luminescence quenching and resistivity lowering. Surprisingly, above this threshold, the photoluminescence (PL) intensity and resistivity increase again with the trend to saturation [29]. This means that the new form of defect forces changes in the location and environment of RE in the ZnO lattice. Nevertheless, because defects shorten the lifetime of devices based on such defective material, and the new form of defects cannot be removed by annealing, the optimal RE fluence applied to one-step implantation should be selected from the range of fluences below this plastic deformation edge.

The most typical lattice recovery is performed using conventional equilibrium thermal processing such as furnace annealing (FA) or rapid thermal annealing (RTA) in a variety of atmospheres such as Ar, and O_2_, as well as in a vacuum [39,40,41,42,43,44]. For ZnO samples implanted with RE ions with fluences below the plastic deformation threshold, such thermal annealing leads to effective recrystallization of the lattice damaged during ion implantation, as well as the relaxation of the out-of-plane lattice strain [38]. However, it is simultaneously associated with the RE atoms’ diffusion, and further, their agglomeration on the surface. As reported in ref. [39], the complete recovery is attained after thermal annealing at 1100 °C, but this apparent success is paid off with dramatic changes in the RE depth profile. The studies have shown that directly after RT-implantation approximately half of the RE ions are located at the substitution position in the ZnO lattice. During thermal annealing, the RE ions become mobile, initially moving to interstitial positions and further toward the sample surface. The RE depth distribution remains almost unchanged for temperatures up to 800 °C, but at 1100 °C all impurity atoms precipitate on the sample surface. This effect should be avoided since the precipitated ions become optically inactive [39].

The above overview accurately shows that the implantation and annealing of ZnO implanted with RE is non-trivial. From the point of view of applications in optoelectronics, an important issue is that, regardless of the technique of incorporation of RE into the crystal matrix, the luminescence quenching effect associated with the RE concentration takes place [49,50,51,52]. Secondly, after annealing, the diffusion effect of RE ions leads to their precipitation on the surface, where they become optically inactive. Therefore, in contrast to many previous works on ZnO implanted with RE, which have focused almost exclusively on the study of a few doses and one selected kind of annealing process, this paper presents a comprehensive study on the optimal implantation and annealing conditions, providing the most efficient luminescence of RE^3+^ ions in the ZnO matrix. We compare deep and shallow implantations, implantations performed with different fluencies and at different high temperatures (HT) as well as at standard RT. A range of post-implantation annealing processes such as rapid thermal annealing (RTA, minute time-duration) at different temperatures, times, and atmospheres (O_2_, N_2_, and Ar), flash lamp annealing (FLA, millisecond time-duration) and plasma pulse annealing (PPA, microsecond time-duration) are investigated as well. Post-implantation damages in the ZnO lattice, structure recovery after annealing, as well as RE depth profiles and their lattice site location in the matrix before and after annealing, were monitored by channeling Rutherford backscattering spectrometry (RBS/c), while the optical response was studied by photoluminescence spectroscopy (PL). The surface morphology was examined by atomic force microscopy (AFM) and scanning electron microscopy (SEM) with an energy-dispersive spectrometry (EDS) system. Most of the studies were performed on ZnO implanted with Yb because the optical response from Yb^3+^ occurs in the infrared (IR) region, where the characteristic, band edge, and defect-related PL emissions of ZnO are not present [17,39].

It is worth mentioning, that experimental PL results on ZnO:RE systems published so far are not fully consistent for samples obtained by different methods (or the same method, but grown under various conditions) [53,54], and also strongly depend on the methods and conditions of RE-doping [55]. Therefore, it is very difficult to complete a comprehensive picture of ZnO:RE systems based on the available literature. The current research aims to fill this gap. We hope that the results presented here will positively contribute to a deeper understanding of such optical systems.

## 2. Material and Methods

Epitaxial ZnO films grown by atomic layer deposition (ALD) at the Institute of Physics, Polish Academy of Science (IP PAS) on the GaN/Al_2_O_3_ substrate at 300 °C were used in the studies. The details of the growth process can be found in ref. [56]. The crystalline quality of the unmodified material was confirmed by XRD and RBS/c studies reported elsewhere [38,57]. The thickness of the deposited layers was about 1 µm.

The ZnO layers were implanted at RT, with a medium-energy 150 keV Yb^+^ ion beam and fluences ranging from 5 × 10^14^ up to 1 × 10^16^/cm^2^ at the Ion Beam Centre, Helmholtz-Zentrum Dresden-Rossendorf (HZDR), Germany. The selected implantation energy allows for obtaining a doped layer less than 100 nm thick. Subsequently, RTA at 800 °C for 10 min in an oxygen atmosphere was performed using an Accu Thermo AW-610 from Allwin21 Corporation equipment at IP PAS. In this system, the temperature of 800 °C is achieved in 20 s while cooling down until RT in a gas flow is achieved within about 300 s. To explore the subject, the set of RT-implanted samples with the 150 keV Yb ions at a fluence of 5 × 10^14^/cm^2^ were also annealed at 800 °C for 10 min in oxygen (O), nitrogen (N), and argon (Ar) atmospheres.

Post-implantation FLA was performed for 20 ms with a flash energy density of 110 J/cm^2^ in an oxygen-rich atmosphere using the FLA system located at HZDR. For PPA an “IBIS” plasma pulse generator operated at the National Centre for Nuclear Research (NCBJ) was employed. This machine produces plasma jets of a duration of about 1 μs (melt time) and an energy density of 1.17 J/cm^2^. Such pulses can melt a thin surface layer of about 0.7 μm of ZnO. The molten layer re-grows epitaxially with a recrystallization rate of ~1 m/s.

Another part of the ZnO layers was HT-implanted with the 150 keV Yb ions at a fluence of 1 × 10^15^/cm^2^ at temperatures of 600, 700, 800, and 900 °C. Additionally, some layers were HT-implanted at 600 °C with energies of 500 keV and 1000 keV, leading to the doped layer thickness of about 150 and 200 nm, respectively. HT implantations were performed at HZDR.

Crystal structure quality, damage recovery, as well as Yb depth profiles, and Yb ions location in the ZnO crystal lattice, were evaluated by RBS/c at the Ion Beam Centre, HZDR, using 1.7 MeV He^+^ ions. The backscattered particles were detected by a silicon surface barrier detector at a backscattering angle of 170° with a depth resolution <5 nm and an energy resolution <20 keV.

The optical properties of RE-implanted and annealed ZnO were studied by PL spectroscopy at the Semiconductor Materials Department, HZDR. The PL spectra were recorded at RT using a Jobin Yvon Triax 550 monochromator and a cooled InGaAs detector for measurements in the IR region. A UV laser with 325 nm wavelength and 8 mW power was used for sample excitation.

For a selected set of samples, the surface microstructure and elemental mapping were examined by a Zeiss EVO^®^ MA10 scanning electron microscope (SEM) with an energy-dispersive spectrometry (EDS) system, located at NCBJ. For the studies the EDX BrukerXFlash Detector 5010 (Bruker Corp., Billerica, MA, USA) and dedicated Quantax 200, Esprit 1.9 code were applied. The SEM-EDS observations were performed for a variety of magnifications and operating voltage (Figures S1–S3). The surface morphology was also investigated by atomic force microscopy (AFM, Bruker Dimension Icon) using silicon nitride probes with sharp tips (a tip radius: 2 nm) in the peak force tapping mode at IP PAS. Surface roughness was determined by a root-mean-square (RMS) roughness of the AFM height measurements from images taken from a 10 × 10 μm^2^ region (Figures S4 and S5).

## 3. Results and Discussion

In many respects, the HT implantation process can be considered an alternative to RT implantation, followed by annealing. To investigate this issue in more detail, the set of samples, implanted at different temperatures (RT, 600, 700, 800, and 900 °C) with 150 keV of Yb ions to the fluence 1 × 10^15^/cm^2^ as well as the sample implanted at RT with the same fluencies and energy of the Yb- ions and subsequently annealed for 10 min at 800 °C in O_2_, were compared.

**Figure 1:**
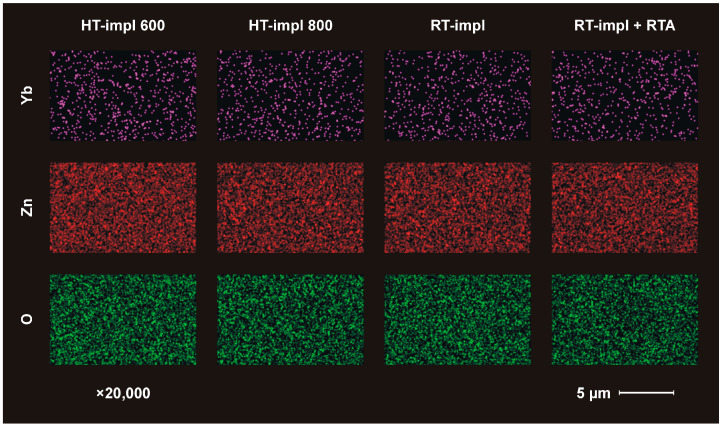
EDS mapping of Yb (pink), Zn (red), and O (green) for ZnO implanted with 150 keV Yb ions to the fluence of 1 × 10^15^/cm^2^ was performed for samples implanted at different temperatures: 600 °C (HT-impl 600), 800 °C. (HT-impl 800), and RT (RT-impl) as well as implanted at RT and subsequently RTA-annealed for 10 min at 800 °C in an oxygen atmosphere (RT-impl +RTA).

The chemical distributions (EDS mapping) of Yb, Zn and O, with 20,000× magnification and an accelerating voltage of 20 kV, are shown in Figure 1. Even though the concentration of Yb in the samples is very small (1 × 10^15^ ions/cm^2^ corresponds to 0.14 at. %), the EDS mapping, collected from a significantly greater depth than the modified implantation layer, detects the Yb signal. As seen in Figure 1, Yb atoms are distributed uniformly over the whole surface sample; however, SEM-EDS does not reveal any subtle differences between the samples prepared in different ways.

**Figure 2:**
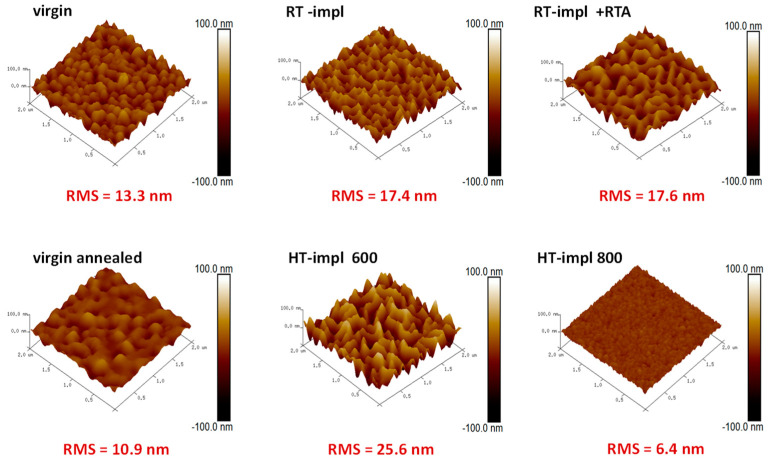
AFM imaging of the surface morphology for ZnO before (virgin) and after implantation at different temperatures with 150 keV Yb ions to the fluence of 1 × 10^15^/cm^2^ as well as RT-implanted samples and subsequently RTA-annealed for 10 min at 800 °C in O_2_.

In turn, the AFM studies presented in Figure 2 show the positive contribution of temperature to the smoothness of the layers. An RMS value of ~13.3 nm is measured for the virgin samples, while for temperature-treated samples this value decreases by up to ~6.4 nm. Any other effects, e.g., the appearance of crystalline precipitation of Yb after temperature treatment in the ZnO films, are not detected.

**Figure 3:**
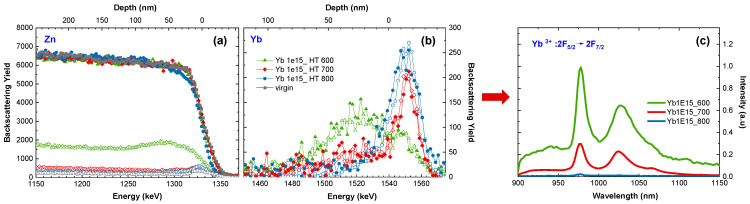
Random (solid symbols) and aligned (open symbols) RBS spectra (**a**,**b**) obtained for ZnO implanted at different temperatures with 150keV Yb ions at the fluence of 1 × 10^15^/cm^2^. The corresponding PL spectra (**c**).

The depth distributions of matrix and impurity atoms were specified by RBS. Moreover, thanks to the use of this method in the channeling mode, the depth distributions of defects caused by ion implantation as well as the Yb lattice site location were also determined. The random and aligned (i.e., taken in the channeling mode) RBS spectra obtained for ZnO before and after HT implantation at different temperatures with 150 keV Yb ions at the optimal fluence of 1 × 10^15^/cm^2^ [29] show separated signals coming from He ions backscattered on Zn and RE atoms (Figure 3). However, differences in the intensity of those signals are significant; therefore, for clarity, they are presented on a different scale (Figure 3a,b). Separate signals mean that crystal lattice defects and RE behavior can be simultaneously monitored, which is an advantage of the RBS/c technique in such analyses. The RBS studies show that with the increase of the implantation temperature, the heights of aligned spectra decrease, which means that the level of defects become smaller with the temperature of implantation. However, at the same time, the RE profile changed, reflecting the diffusion of RE ions toward the sample surface. For implantation at 800 °C, the perfect crystal structure recovery is observed (Figure 3a), but most of the RE ions precipitate on the surface, which results in a high RBS signal at an energy of about 1550 keV (Figure 3b).

Comparing the aligned spectra with the random one for the Yb signal observed in the energy region 1470–1570 keV, it can be remarked that for HT-implanted samples, the random and aligned RBS spectra of RE presented in Figure 3b have the same intensity, which means that the RE ions are not incorporated into the ZnO matrix.

The details of these observations can be listed numerically by the minimum yield (χ_min_) and substitutional fraction (f_s_) parameters [34] (Table 1). The χ_min_ is the ratio of the backscattering yield of an aligned spectrum (calculated for a selected energy range) to the corresponding yield of the random one. It represents the level of structural defects in the Zn sublattice. The f_s_ is defined as the relative amount of impurity atoms occupying lattice site positions. Table 1 specifies also the maximum value of the backscattering yield in the Yb peak, located around 1550 keV, which reflects the change in the amount of Yb ions precipitated on the surface (Figure 3b), as well as the PL intensity at 978 nm (Figure 3c).

Structural changes directly affect the optical properties of the samples. A typical PL spectrum of Yb ions in the 3+ oxidation state (Figure 3c), observed in the IR spectral range between 900 and 1150 nm, is not affected by ZnO characteristic emissions, coming from the near band edge and deep-level emission connected with defects, that appear in the visible region [17]. The IR PL emission from Yb^3+^ observed at about 980 nm corresponds to the main radiative transition from the excited ^2^F_5/2_ energy state to the ^2^F_7/2_ ground state of Yb^3+^. Broad peaks located between 1000 and 1150 nm correspond to the Yb^3+^ ion emission vibrionic band (^2^F_5/2_→^2^F_7/2(n)_; n = 1,2,3,4) [58]. In contrast to RT implantation, the RE-ions after HT implantation are optically active (Figure 3c). However, as the implantation temperature increases to 700 °C, the luminescence coming from RE decreases drastically, and finally, for 800 °C, when all RE ions precipitate on the surface, the luminescence coming from Yb^3+^ completely disappears.

**Figure 4:**
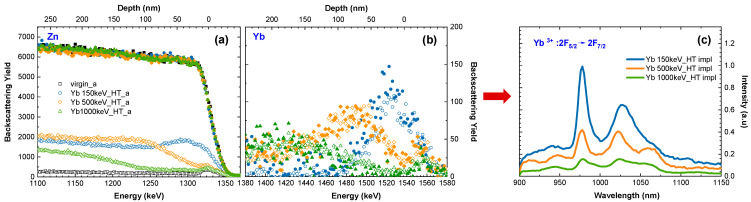
Random (solid symbols) and aligned (open symbols) RBS spectra (**a**,**b**) obtained for ZnO implanted with different energies of Yb ions at the fluence of 1 × 10^15^ /cm^2^ at 600 °C. The corresponding PL spectra (**c**).

Other interesting conclusions have been drawn from the experiment, in which different energies of implanted ions are used, resulting in a different implantation depth. In the experiment, epitaxial ZnO films were implanted at 600 °C with Yb ions at energies of 150, 500, and 1000 keV, which results in ~100, 160, and 240 nm modified layers, respectively. The RBS-aligned spectra (Figure 4a,b), confirmed by simulations performed using the McChasy code [59,60], show that the number of point defects increases with the implantation energy, but the density of extended defects, such as dislocations, is smaller for the faster ions. Regardless of these subtle differences in the defects’ structure, the luminescence efficiency, presented in Figure 4c, is significantly lower for deeper implantations, probably due to the high-light absorption coefficient, which for ZnO is about 5 × 10^4^/cm [61].

To eliminate the undesirable RE out-diffusion effect, alternative annealing techniques are tested. The millisecond range FLA has already been successfully used to improve the crystalline quality of RT-implanted materials [62,63]. Furthermore, in the case of ZnO:RE implanted at RT with fluences below the plastic deformation threshold, the RE out-diffusion effect after FLA is not observed, and RE ions are mainly placed at substitutional positions. However, unfortunately, the RE ions after FLA remain optically inactive [39].

**Figure 5:**
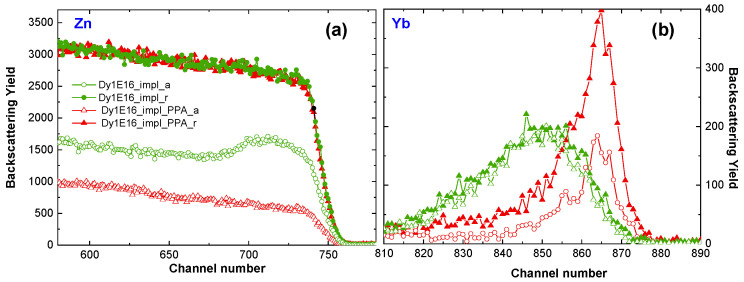
(**a**,**b**) Random and aligned RBS spectra obtained for ZnO implanted with 150 keV Dy ions to the fluence of 1 × 10^16^/cm^2^ and subsequently annealed using plasma pulse annealing with a pulse energy of 1.17 J/cm^2^.

The crystalline structure recovery effect of ZnO:RE implanted at RT is also observed after PPA, as shown in Figure 5a, where the RBS spectra of ZnO implanted at RT with Dy ions and subsequently PPA annealed are presented. The above studies are conducted on the ZnO:Dy system; however, as previously reported, there is no difference in the level of structural damage after implantation with different RE ions at the same fluence [17]. Unfortunately, after this kind of annealing, the RE atoms diffuse to the sample surface (Figure 5b), and no light coming from RE^3+^ is observed, as in the high-temperature cases (see Figure 3c). This means that neither FLA nor PPA are useful for the optical activation of RE in ZnO:RE systems.

**Figure 6:**
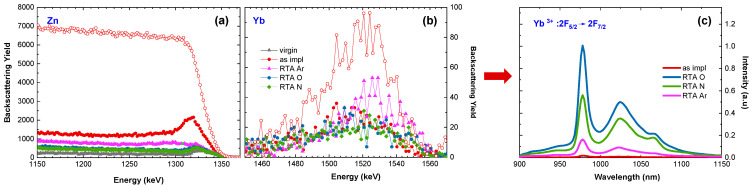
Random (open symbols) and aligned (solid symbols) RBS spectra (**a**,**b**) obtained for ZnO implanted at RT with 150 keV Yb ions to the fluence 5 × 10^14^ /cm^2^ and subsequently RTA-annealed for 10min at 800 °C in different atmospheres. The corresponding PL spectra (**c**).

**Figure 7:**
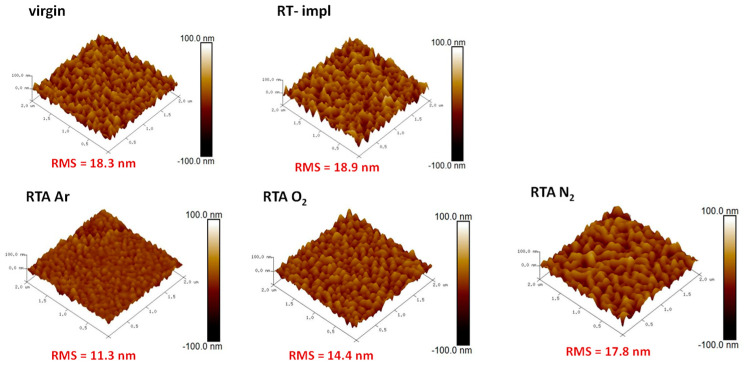
AFM imaging of the surface morphology for ZnO implanted with 150 keV Yb ions to the fluence 5 × 10^14^/cm^2^ and subsequently RTA-annealed for 10 min at 800 °C in different atmospheres N_2_, Ar, O_2_.

In the last part of the study, the optimal atmosphere of RTA post-RT implantation annealing is tested. A comparison of RBS-aligned spectra obtained for ZnO implanted at RT with a fluence of Yb ions below the plastic deformation threshold and subsequently annealed for 10 min at 800 °C in O_2_, N_2_, and Ar atmospheres is presented in Figure 6a,b. It has been reported that too long annealing time leads to an increase in the number of RE atoms on the surface, and thus to a decrease in PL efficiency. The 10 min RTA annealing time results in a balance between structure recovery and RE out-diffusion [39]. As can be seen in Figure 6a,b, and Table 2 as well, the spectra and all parameters vary significantly depending on the annealing atmosphere. That applies to both the thermal-induced structural recovery of ZnO and the migration of Yb to the interstitial site as well. The recovery level, as well as the Yb behavior after the RTA annealing in oxygen and nitrogen atmospheres, are very similar. The weakest recovery is observed after annealing in the Ar atmosphere. In turn, the luminescence response, to these structural changes, shows the most efficient luminescence for oxygen annealing, as presented in Figure 4c. Interestingly, the PL intensity is not directly correlated with the RMS value, which is 14.4 nm for oxygen annealing so between the values obtained for Ar and N_2_ annealing (Figure 7).

**Figure 8:**
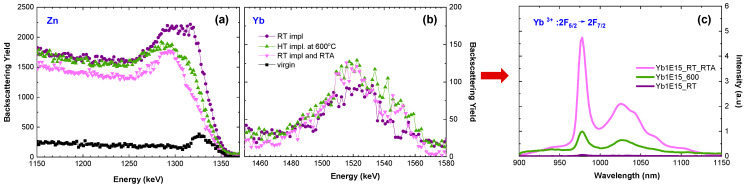
Aligned RBS spectra (**a**,**b**) obtained for ZnO implanted with 150 keV Yb ions to the fluence of 1 × 10^15^/cm^2^ at 600 °C and RT and subsequently RTA-annealed for 10 min at 800 °C in an oxygen atmosphere. The corresponding PL spectra (**c**).

Finally, the optical response of Yb ions implanted at the optimum temperature (600 °C) is compared with the optical response of these ions implanted at RT and subsequently annealed at 800 °C in oxygen (Figure 8c). Although, as can be observed in the aligned RBS spectra (and Table 3), the crystal structure recovery is only slightly better for RT implantation and subsequent annealing (Figure 8a), the recorded luminescence efficiency for this sample is four times higher (Figure 8c), showing that this way of preparing samples is the most beneficial for the optical applications of ZnO:RE systems. This effect might be assigned to both better crystal structure recovery and a lower number of Yb ions on the crystal’s surface as can be deduced from Figure 8b. However, the role of oxygen in the luminescence of ZnO seems to be important. It may be assumed that oxygen diffuses into the sample during RTA and oxidizes RE to the 3+ state which increases PL [39], but the other scenarios are also possible [64].

## 4. Summary

The effects of implantation and subsequent annealing conditions on the surface morphology, defects in ZnO lattice and RE locations, as well as on the optical response coming from Yb ions in the 3+ oxidation state were studied systematically. The results of deep and shallow implantations, performed at high and room temperature, as well as a range of post-RT-implantation annealing processes were compared for ZnO:RE systems.

It is found that shallow implantation (with the energy of RE ions about 150 keV) with the RE ion fluence below the plastic deformation edge of ZnO crystals (less than 1.5 × 10^15^/cm^2^) is optimal for optical applications of ZnO:RE systems. It was also established that RE ions after HT implantation at a temperature lower than 700 °C are optically active; however, the temperature of 600 °C is optimal, ensuring a higher luminescence efficiency of Yb^3+^. In contrast, directly after RT implantation most of the Yb ions are optically inactive; therefore, post-implantation annealing is necessary. For the samples implanted at RT, the FLA, PPA, and RTA thermal annealing in O_2_, N_2_, and Ar atmospheres have been tested for RE activation. The highest luminescence efficiency of RE^3+^ was obtained after 10 min RTA annealing performed at 800 °C in oxygen. The comparison of the optical response of this sample with the ZnO:Yb system implanted at 600 °C with the same fluence and energy, shows over four times higher luminescence efficiency of the former system. The light emission from the ZnO:RE system prepared in the above way is so bright that it can be observed by the naked eye, which makes the ZnO:RE systems worth considering for applications in LED or display technologies.

## Figures and Tables

**Table 1:** Numerical evaluation of the RBS and PL spectra of ZnO implanted at different high temperatures with 150 keV Yb ions at the fluence of 1 × 10^15^/cm^2^.

	RBS			PL
	χ_min_ (%)	f_s_ (%)	The Backscattering Yield of Yb Ions on the Surface (Counts)	Intensity (a.u.)
virgin	3.2	-	-	-
HT-impl 600	27.0	20.6	88	1.00
HT-impl 700	6.6	5.6	205	0.30
HT-impl 800	4.2	3.5	248	0.03

**Table 2:** Numerical evaluation of the RBS and PL spectra of ZnO implanted at RT with 150 keV Yb ions at the fluence of 5x10^14^ /cm^2^ and RTA-annealed.

	RBS			PL
	χ_min_ (%)	f_s_ (%)	The Backscattering Yield of Yb Ions on the Surface (Counts)	Intensity (a.u.)
Virgin	2.9	-	-	-
RT-impl	20.4	64.9	7	0.02
RT-impl+RTA O_2_	6.2	40.1	20	1.00
RT-impl+RTA N_2_	5.6	55.9	16	0.56
RT-impl+RTA Ar	12.0	16.5	20	0.16

**Table 3:** Numerical evaluation of the RBS and PL spectra of ZnO implanted at RT and 600 °C with 150 keV Yb ions at the fluence of 1 × 10^15^/cm^2^.

	RBS			PL
	χ_min_ (%)	f_s_ (%)	The Backscattering Yield of Yb Ions on the Surface (Counts)	χ_min_ (%)
virgin	3.2	-	-	-
HT 600	27.0	20.6	88	1.00
RT	28.6	49.1	25	0.04
RT+RTA	23.7	26.5	64	4.59

## Data Availability

Not applicable.

## Supplementary Materials

The following supporting information can be downloaded at: https://www.mdpi.com/article/10.3390/ma16051756/s1, Figure S1: SEM imaging of the surface morphology with a variety of magnifications and operating voltage for ZnO implanted at 800 °C with 150 keV Yb ions to the fluence of 1 × 10^15^/cm^2^; Figure S2: EDS mapping with a variety of magnifications and operating voltage for ZnO implanted at 800 °C with 150 keV Yb ions to the fluence of 1 × 10^15^/cm^2^; Figure S3: SEM imaging of the surface morphology for ZnO implanted with 150 keV Yb ions to the fluence of 1 × 10^15^/cm^2^ at different temperatures: 600 and 800 °C and RT and subsequently RTA-annealed for 10 min at 800 °C in an oxygen atmosphere; Figure S4: AFM imaging of the surface morphology for ZnO implanted with 150 keV Yb ions to the fluence of 1 × 10^15^/cm^2^ at different temperatures: 600 and 800 °C and RT and subsequently RTA-annealed for 10 min at 800 °C in an oxygen atmosphere; Figure S5: AFM imaging of the surface morphology for ZnO implanted with 150 keV Yb ions to the fluence 1 × 10^15^/cm^2^ and subsequently RTA-annealed for 10min at 800 °C in different atmospheres N_2_, Ar, O_2_.
