# Supplementary material for: Enhanced Luminescence of Yb3+ Ions Implanted to ZnO through the Selection of Optimal Implantation and Annealing Conditions
Source: Materials (Basel). 2023 Feb 21;16(5):1756. doi: 10.3390/ma16051756 (PMC10003926; doi:10.3390/ma16051756)

Figure S1. SEM imaging of the surface morphology with a variety of magnifications and operating voltage *for ZnO implanted at 800℃ with 150 keV Yb ions to the fluence of* 1×10^{15} */cm*^{2}

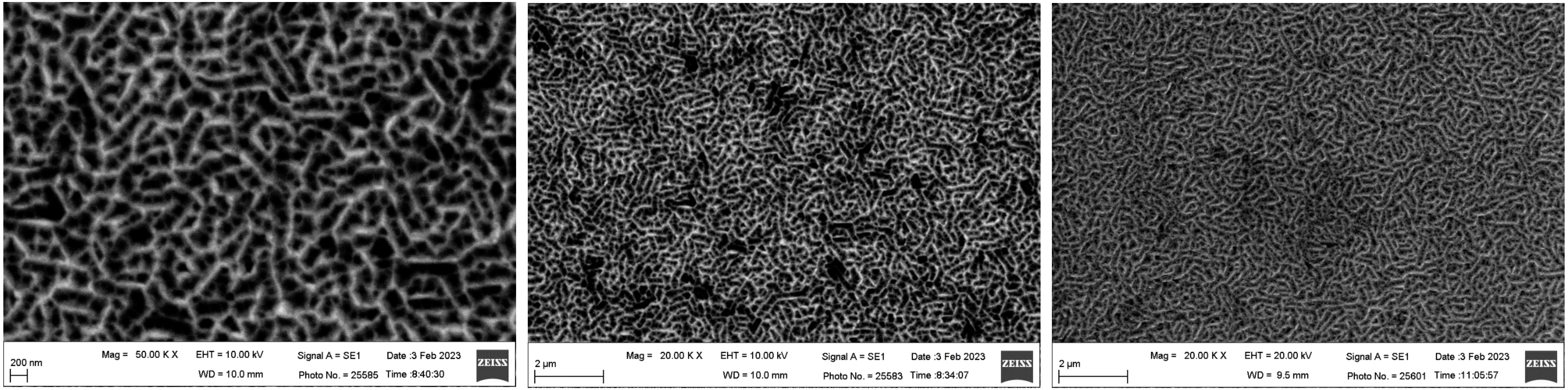

Figure S2. EDS mapping with a variety of magnifications and operating voltage *for ZnO implanted at 800℃ with 150 keV Yb ions to the fluence of* $1x10^{15}$ */cm*^{2}

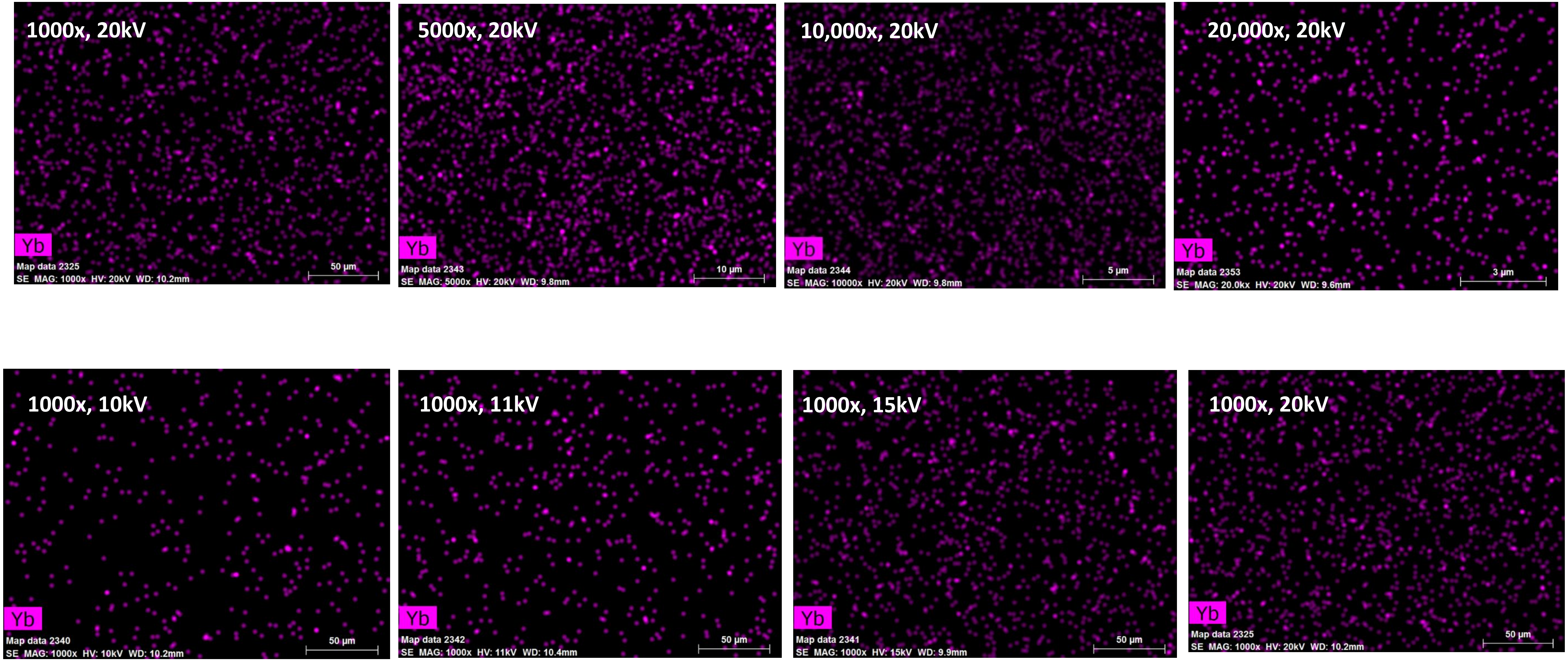

Figure S3. SEM imaging of the surface morphology *for ZnO implanted with 150 keV Yb ions to the fluence of* $1x10^{15}$ */cm*^{2} *at different temperatures: 600 and 800 ℃ and RT and subsequently RTA-annealed for 10min at 800 ℃ in an oxygen atmosphere*

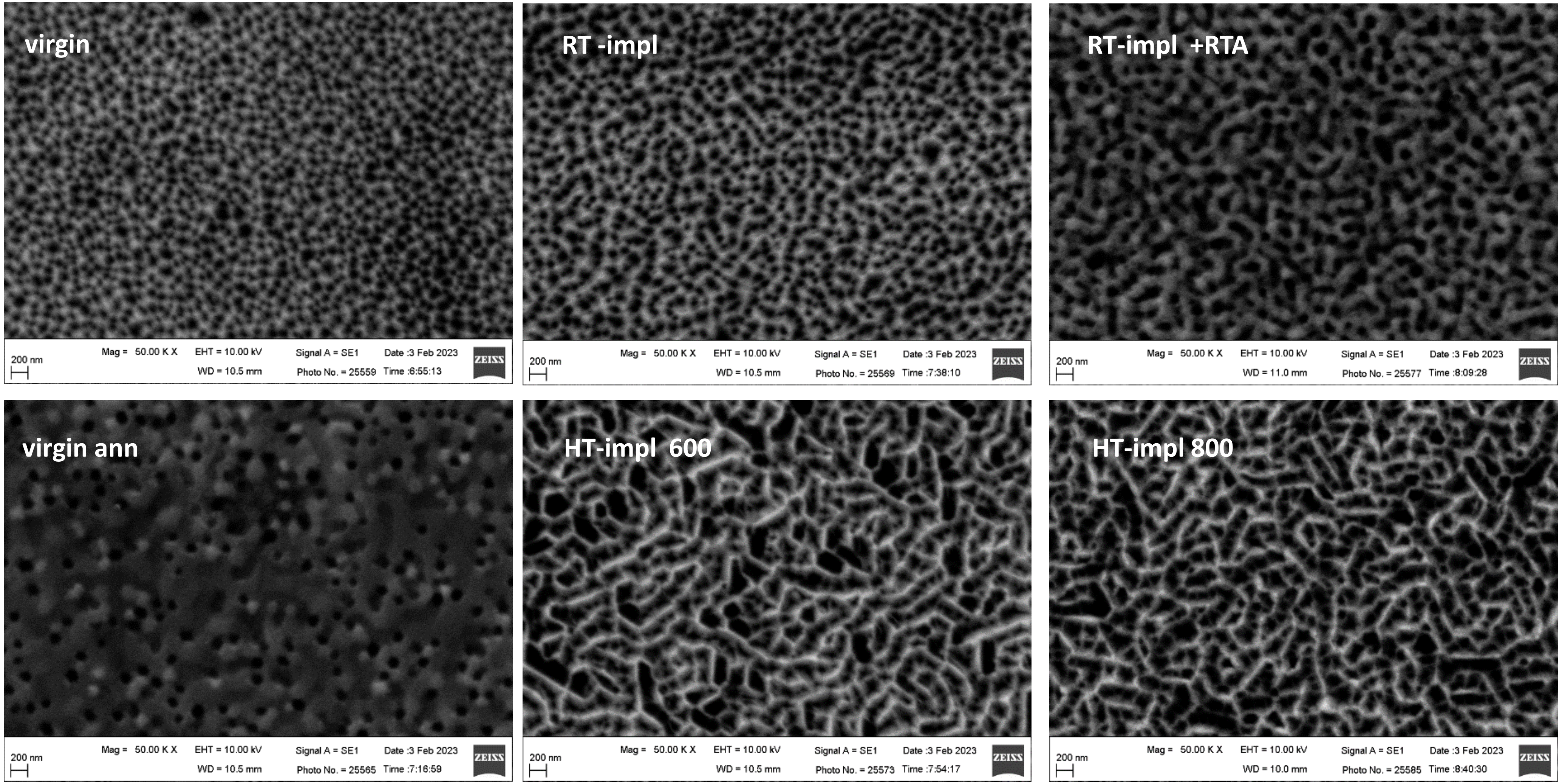

Figure S4. AFM imaging of the surface morphology *for ZnO implanted with 150 keV Yb ions to the fluence of 1x10*^{15} */cm*^{2} *at different temperatures: 600 and 800 ℃ and RT and subsequently RTA-annealed for 10min at 800 ℃ in an oxygen atmosphere*

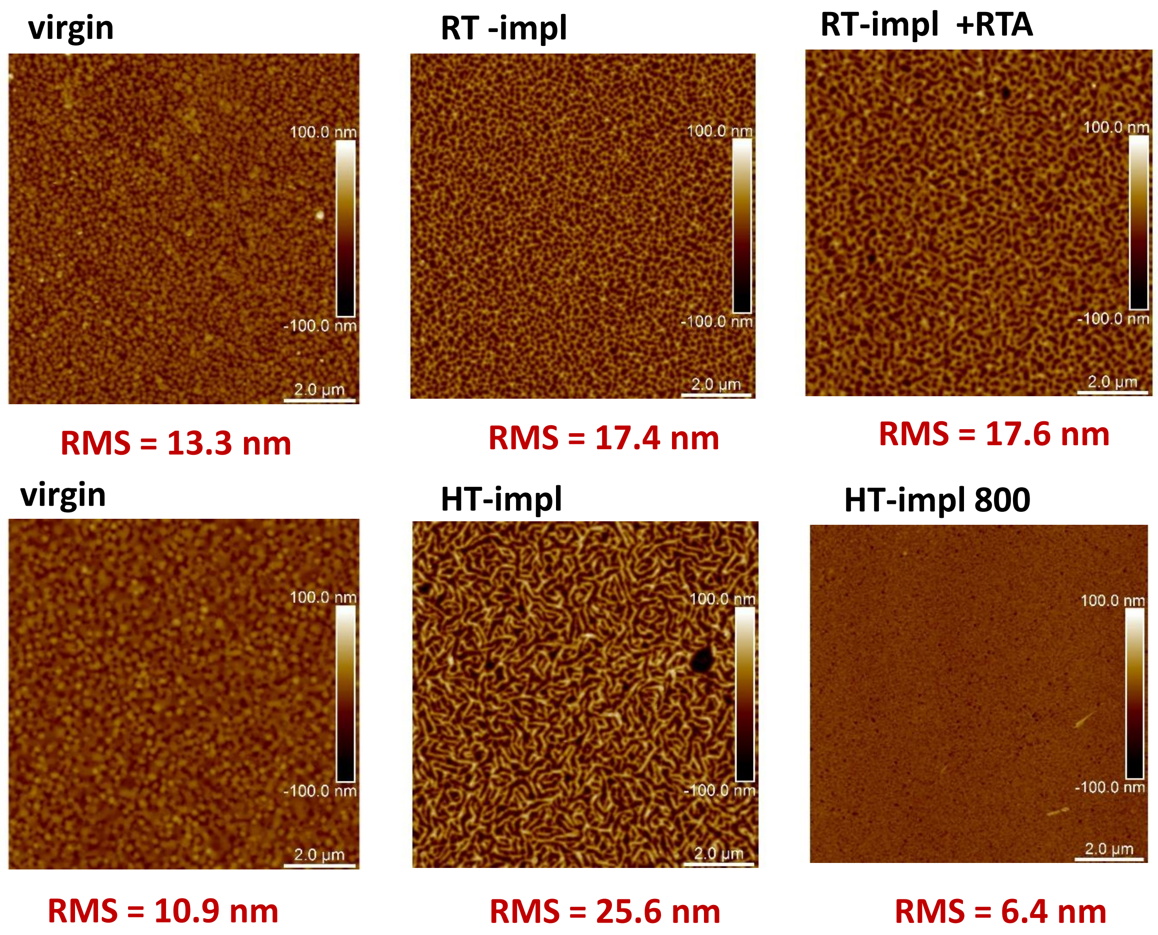

Figure S5. AFM imaging of the surface morphology *for ZnO implanted with 150 keV Yb ions to the fluence 1x10*^{15} */cm*^{2} *and subsequently RTA-annealed for 10min at 800 ℃ in different atmospheres* N_2*, Ar,* O_2*.*

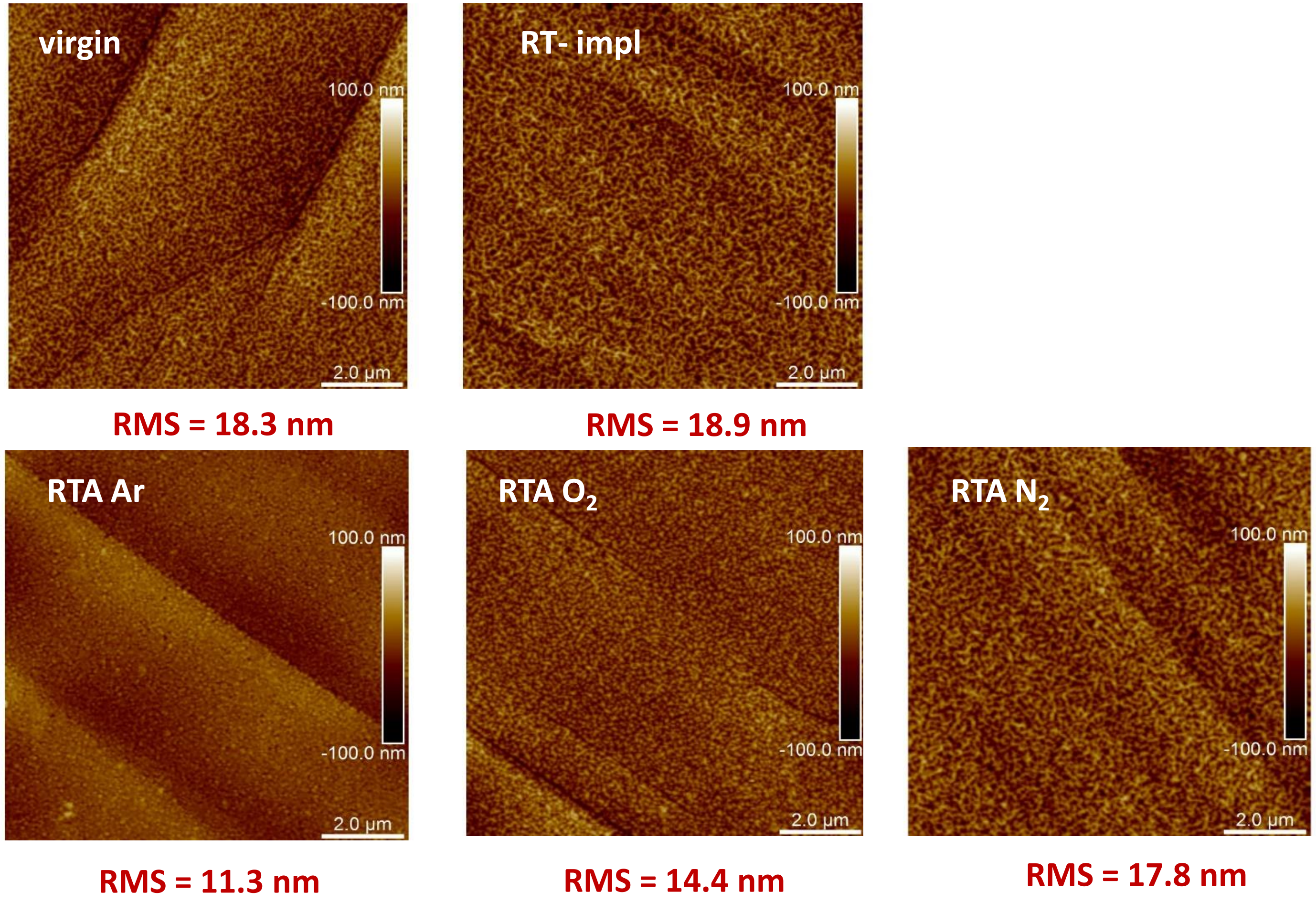

Supplement: Supplementary file 1 [file materials-16-01756-s001.zip › materials-2181920-supplementary.pdf]
